# Solitary beam propagation in periodic layered Kerr media enables high-efficiency pulse compression and mode self-cleaning

**DOI:** 10.1038/s41377-021-00495-9

**Published:** 2021-03-10

**Authors:** Sheng Zhang, Zongyuan Fu, Bingbing Zhu, Guangyu Fan, Yudong Chen, Shunjia Wang, Yaxin Liu, Andrius Baltuska, Cheng Jin, Chuanshan Tian, Zhensheng Tao

**Affiliations:** 1 State Key Laboratory of Surface Physics and Department of Physics, Fudan University, Shanghai, China; 2 Institute of Photonics, TU Wien, Gusshausstrasse 27/387, Vienna, Austria; 3 Department of Applied Physics, Nanjing University of Science and Technology, Nanjing, Jiangsu 210094 China

**Keywords:** Ultrafast lasers, High-harmonic generation, Nonlinear optics

## Abstract

Generating intense ultrashort pulses with high-quality spatial modes is crucial for ultrafast and strong-field science and can be achieved by nonlinear supercontinuum generation (SCG) and pulse compression. In this work, we propose that the generation of quasi-stationary solitons in periodic layered Kerr media can greatly enhance the nonlinear light-matter interaction and fundamentally improve the performance of SCG and pulse compression in condensed media. With both experimental and theoretical studies, we successfully identify these solitary modes and reveal their unified condition for stability. Space-time coupling is shown to strongly influence the stability of solitons, leading to variations in the spectral, spatial and temporal profiles of femtosecond pulses. Taking advantage of the unique characteristics of these solitary modes, we first demonstrate single-stage SCG and the compression of femtosecond pulses from 170 to 22 fs with an efficiency >85%. The high spatiotemporal quality of the compressed pulses is further confirmed by high-harmonic generation. We also provide evidence of efficient mode self-cleaning, which suggests rich spatiotemporal self-organization of the laser beams in a nonlinear resonator. This work offers a route towards highly efficient, simple, stable and highly flexible SCG and pulse compression solutions for state-of-the-art ytterbium laser technology.

## Introduction

Generating high-energy, ultrashort pulses with high-quality spatial modes is essential for numerous applications in ultrafast optics and strong-field physics. Although the remarkable development of the optical parametric chirped-pulse amplification (OPCPA) technique has enabled the direct generation of ultrashort pulses^1–3^ with high average power^4^ and peak power^5^, its picosecond pump design remains very sophisticated and inaccessible to many laboratories. For the existing large number of ultrafast lasers in physics, femtochemistry and femtobiology labs, a simple and reliable scheme for external pulse compression is in demand. Another route to shorter pulse durations externally is through nonlinear pulse compression, which relies on supercontinuum generation (SCG) enabled by self-phase modulation (SPM) in a *χ*^(3)^ nonlinear medium, in combination with a negative dispersive delay line for phase compensation^6^.

In past years, great efforts have been made to manipulate the characteristics of femtosecond pulses and to engineer nonlinear media for SPM to achieve sustainable light-matter interactions and to offer ultra-broadband SCG. For instance, with the spatial mode confined by gas-filled hollow-core fibres (HCFs), femtosecond pulses can interact with a gas medium over several metres, enable the compression of pulses with high energy^7,8^ and high average power^9^, and provide high compression ratios^10^. However, the HCF technique has several drawbacks: First, it is sensitive to alignment, and pointing-stabilization setups are usually required for stable performance. Second, it is difficult to maintain because of the vacuum systems and gas-vacuum interfaces. Third, the fibre-coupling efficiency is usually low (~60%)^6,9^, although higher efficiency has recently been reported by implementing large-diameter capillaries^10,11^ and photonic crystal fibres^12,13^. More recently, the gas-filled multi-pass cell has achieved long-distance light-gas interactions with an efficiency of ~90%^14–18^. However, in addition to the inconvenient vacuum systems, because of the large number of reflections on the cavity mirrors, it requires a state-of-the-art coating with low group-delay dispersion (GDD) and high reflectivity, which is costly and technically challenging. We note that the key for high-quality SCG is to realize the long-distance propagation of intense femtosecond pulses with stable profiles in both space and time under the influence of Kerr nonlinearity, which, in fact, can be accomplished by soliton formation^19^.

Compared to gas media, SCG in condensed media is advantageous because it is simple, flexible and robust, allowing for free-space setups. Recently, SCG with multiple thin plates of Kerr media (multiplate SCG) has attracted much attention^20–25^. By placing the beam self-focusing in the free space between material plates, this technique elaborately circumvents the optical breakdown when the peak power is higher than the critical power *P*_cr_^19^, allowing substantial enhancement of the nonlinear Kerr interaction^21,23^. Although the generation of few-cycle pulses has been demonstrated with a two-stage compressor^24^, the existing implementations of multiplate SCG and pulse compression have several limitations because the nonlinear propagation of the beam is still not well controlled. First, complicated space-time coupling leads to strong conical emission, which can cause energy loss >40%^23^. Second, because of the space-time-coupling-induced higher-order dispersion, the dispersion compensation requires custom-designed chirped mirrors or pulse shapers in some cases^23,24^, and strong pedestals can be occasionally observed in the compressed pulses^24^. Finally, the plate thickness and their positions have only been empirically determined thus far, making it difficult to systematically study and repeat.

Here, we point out that it is possible to form solitons in periodic layered Kerr media (PLKM), which can support sustainable light-matter interactions in condensed media. The theory has previously predicted the existence of quasi-stable^26,27^ or solitary spatial modes^28^ in PLKM without considering the space-time coupling. Experimentally, a periodic variation of the beam profiles for *P* ≈ 6 *P*_cr_ has been observed without spatial beam collapse^29^. Ideally, this periodic propagation can be regarded as transverse [(2 + 1)D] discrete spatial solitons, with a repetitive spatial mode on the material layers^28,30^. The stability of these solitons, however, could be strongly influenced by space-time coupling. Unfortunately, to date, no experimental studies on the influence of spatiotemporal propagation exist. Neither have their potential applications for SCG and pulse compression been studied.

In this work, we experimentally characterize the space-time coupled propagation of femtosecond pulses with peak power reaching gigawatt in PLKM and successfully identify the formation of quasi-stationary solitons under a range of different conditions. By comparing the experimental and theoretical results, we reveal a universal relationship between the characteristic transverse-mode size and the critical nonlinear phase of the solitary modes, under which the propagation of light wave-packets is localized in both space and time. We also show that the formation and breakdown of solitons are manifested by the correlated variations in the spectral, spatial and temporal profiles of femtosecond pulses. In practice, we demonstrate two applications. First, taking advantage of these space-time localized solitary modes, we can significantly enhance the nonlinear light-matter interaction and suppress the spatial and temporal losses, achieving ~8-fold pulse compression with >85% efficiency in a single-stage compressor. The spatiotemporal quality of the compressed pulses is further confirmed by the nonlinear process of high-harmonic generation (HHG), which leads to substantial enhancement of the HHG cut-off energy and brightness. Second, spatial mode self-cleaning with high efficiency is demonstrated under the solitary modes, which can be attributed to the spatial self-organizing effect in a nonlinear resonator.

It is worth noting that with the great advancement in the last decade, Yb-based ultrafast lasers^31–33^ have become very popular in ultrafast physics, femtochemistry and femtobiology laboratories because they exhibit exceptional thermal efficiency and a power-scaling capability, are low in cost and are highly flexible in adjusting pulse energies and repetition rates. However, the pulse durations from these lasers are usually not shorter than 100 fs or even 1 ps; hence, external pulse compression is required for applications. The solitary modes identified in this work can provide a highly efficient, simple, stable and highly flexible solution for the SCG and pulse compression of these lasers. Compared to HCFs and multi-pass gas cells, our method does not require vacuum/gas or pointing-stabilization systems. On the other hand, compared to the previous implementations of multiplate compression, our method exhibits high efficiency and excellent spatiotemporal quality and is highly reproducible because it can be constructed with much fewer degrees of freedom.

## Results

### Stability regions of a nonlinear resonator

**Fig. 1:**
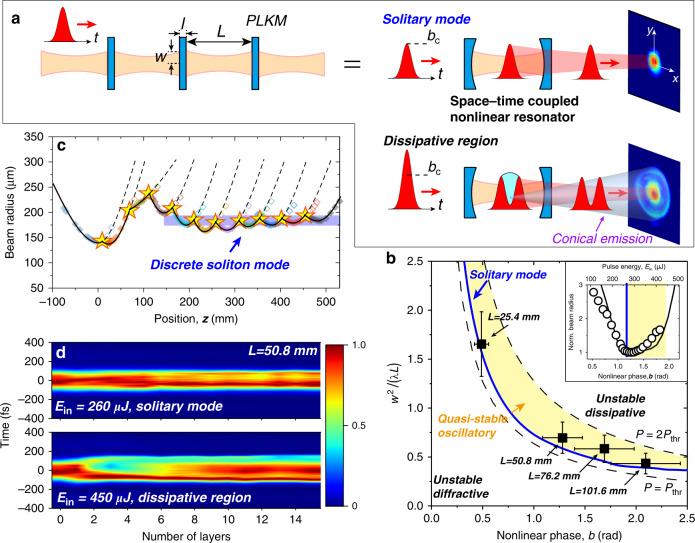
Formation of spatial solitons in PLKM. **a** Illustration of the repetitive self-focusing and diverging pattern in PLKM, which is equivalent to a space-time coupled nonlinear optical resonator. The space-time coupled propagation of femtosecond pulses for the solitary modes and in the dissipative region is presented. When the propagation is in the dissipative region, strong conical emission is contributed by the optical energy at the temporal centre of the pulse, leading to temporal pulse splitting. **b** Universal relationship of the normalized beam radius squared, i.e., *w*^2^/*λL*, as a function of the nonlinear phase *b* for the resonator solitary modes (solid blue line). The dashed lines represent the boundaries for *P*_thr_ < *P* < 2*P*_thr_. The symbols are the experimental results under different resonator lengths. The yellow shaded area is the “quasi-stable oscillatory region”. Inset: Variation in the far-field beam radius as a function of the applied nonlinear phase and incident pulse energy for *L* = 50.8 mm and *b*_c_ = 1.2. **c** Experimentally measured beam radii through propagation in the PLKM with *L* = 50.8 mm and *b*_c_ = 1.2 under the resonant condition. The beam radius in each layer is indicated by the star symbols. **d** Evolution of the temporal intensity profiles as the pulses propagate through the PLKM under the solitary mode (*E*_in_ = 260 μJ) and in the dissipative region (*E*_in_ = 450 μJ)

The repetitive propagation of a high-intensity laser beam in PLKM can be analogous to a cavity resonator with intensity-dependent non-spherical (Kerr-lens) mirrors (see Fig. 1a). Because of the complexity of the spatiotemporal effects introduced by Kerr nonlinearity^19^, the nonlinear Schrödinger equation (NLSE) simulation relies heavily on numerical analysis, which complicates the understanding of the fundamental physical processes. Here, we first resort to the Fresnel-Kirchhoff diffraction (FKD) integral to identify the self-consistent stationary modes. Assuming that the normalized amplitude of the incident optical field is *U*_1_, we find that the amplitude, after propagating through a unit of the resonator and immediately before the next period, is given by

1 $$U_2\left( \rho \right) = - 2\pi je^{j\pi \rho ^2}\mathop{\int}\nolimits_{0}^{\infty} {U_1\left( {\rho {^\prime}} \right){e}^{jb\left| {U_1\left( {\rho {^\prime}} \right)} \right|^2} \cdot {e}^{j\pi \rho {^\prime}^2}J_0\left( {2\pi \rho {^\prime}\rho } \right)\rho {^\prime}{\mathrm{d}}\rho {^\prime}}$$

where *ρ* and *ρ*ʹ are the radial coordinates, rescaled by$\sqrt {\lambda L}$, and *J*_0_ is the zeroth-order Bessel function. Here, one period of the resonator contains a layer of Kerr medium with a thickness of *l* and a subsequent layer of free space with length *L*. In Eq. (1), *b* represents the nonlinear phase given by $b = \frac{{2\pi }}{\lambda }n_2lI_0$, where *I*_0_ is the field intensity. The Fox-Li iteration is then used to numerically find the stationary modes^34^, the nonlinear phase associated with which is defined as the critical nonlinear phase *b*_c_. As shown in Fig. 1b, the characteristic properties of these stationary modes are determined by the transverse-mode radius (*w*) on the layers, the resonator length (*L*) and the critical nonlinear phase (*b*_c_). Here, we define a Fresnel-number-like radius squared, i.e., *w*^2^/*λL*, for convenience of discussion.

For our experimental studies, we employ an Yb:KGW amplifier laser system with a pulse duration Δ*τ* = 170 fs at *λ* = 1030 nm. Transform-limited (TL) femtosecond pulses with p polarization are focused into the PLKM (see Methods and Supplementary Materials (SM) for the experimental setup). The PLKM are composed of polycrystalline Al_2_O_3_ thin plates as the Kerr medium, placed at the Brewster angle to minimize the loss on each plate to <0.5%. The nominal thickness of the plates is fixed at 400 μm (see SM), and the distance between the neighbouring plates equals the resonator length *L*. By adjusting the incident pulse energy, the resonant modes are experimentally identified. The evolution of the spatial beam profiles inside the PLKM is monitored with a 4-*f* imaging setup, while the temporal intensity profiles are measured by second-harmonic-generation frequency-resolved optical gating (SHG-FROG)^35^. We note that we have investigated resonators constructed with Al_2_O_3_, SiO_2_ (fused silica) and CaF_2_ with thicknesses ranging from 100 to 500 μm in our experiments and found that the relationship in Fig. 1b is generally valid.

First, we present findings on the key features of soliton formation and stability. Under a specific resonator length *L* (e.g., *L* = 50.8 mm in the inset of Fig. 1b), we observe a reduction in the far-field beam size and an improvement in the beam quality (see SM) as the incident pulse energy (*E*_in_) approaches a critical value. When the pulse energy is low and the self-focusing in the media is weak, the beam propagation is dominated by diffraction, leading to a diverging beam size on the successive layers (*unstable diffractive region* in Fig. 1b). In contrast, when the incident beam reaches the critical nonlinear phase (*b*_c_) and produces sufficient self-focusing to appropriately balance the diffraction (*E*_in_ = 260 μJ for *L* = 50.8 mm, corresponding to a field intensity of 5.0 × 10^12^ W/cm^2^), the laser beam can repetitively propagate through the PLKM with a well-confined beam size. Indeed, with the 4-*f* imaging measurements, we observe that the transverse beam radius on the material layers rapidly relaxes to a stable value after the self-adjustment in the first few layers under the resonant condition (Fig. 1c). This result clearly demonstrates the formation of discrete spatial solitons^28^. We investigated the formation of solitons in PLKM resonators with different *L* values, and the critical pulse energy ranged from 100 to 600 μJ (see SM). In Fig. 1b, we summarize the experimental results, which exhibit excellent agreement with the FKD model. The agreement here indicates that the formation of spatial solitons is fundamentally caused by the balance between diffraction and nonlinear self-focusing. Moreover, since the temporal profiles are not considered in the FKD model, this agreement also suggests that the femtosecond pulses should have a stable temporal profile throughout the propagation under the solitary modes. This idea is confirmed by the direct FROG measurement of the output pulse profiles after each layer, as shown in Fig. 1d. As a result, we can define these solitary modes as *temporally confined spatial solitons*.

As shown in the inset of Fig. 1b, when *E*_in_ rises above the critical value, the beam size does not immediately diverge. According to the FKD model, this corresponds to a region where the beam size oscillates as it propagates through the PLKM (*quasi-stable oscillatory region*, see SM). The upper boundary of this region is approximately *P* = 3*P*_thr_, where *P*_thr_ is given by *P*_thr_ = *P*_cr_*γn*_0_^27^. The geometrical factor *γ* is given by $\gamma = \frac{L}{l}$. However, we find that the width of this region is generally narrower in the experiments than in the FKD results. This outcome could be attributed to the temporal pulse splitting above the resonance (see below), which is not considered in the model. Beyond 2*P*_thr_, the strong Kerr lens breaks the balance between self-focusing and diffraction, and the beam size grows out of limit within a few periods of propagation (*unstable dissipative region* in Fig. 1b).

**Fig. 2:**
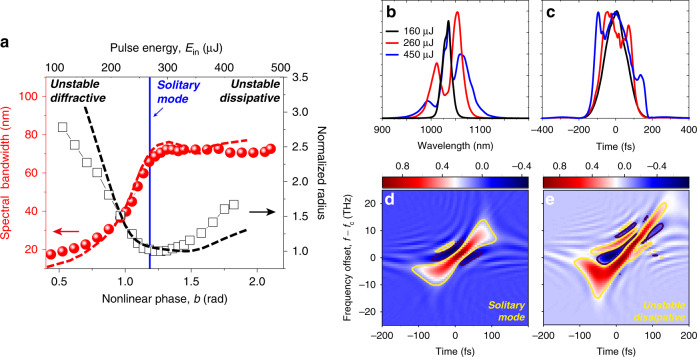
Variations of the spatial, spectral and temporal profiles under different conditions. **a** The spectral bandwidth and normalized beam radius as a function of the nonlinear phase and pulse energy for the PLKM resonator with *L* = 50.8 mm and 15 layers. The dashed lines are the results of the NLSE simulations. **b**, **c** Far-field spectral and temporal profiles in different stability regions. **d** Time-frequency analysis (Wigner plots) of the output pulses under the solitary mode. *f*_c_ is the central frequency. The contours label the 1/e^2^ intensity. **e** Same as (**d**). but for the output pulses in the dissipative region

**Fig. 3:**
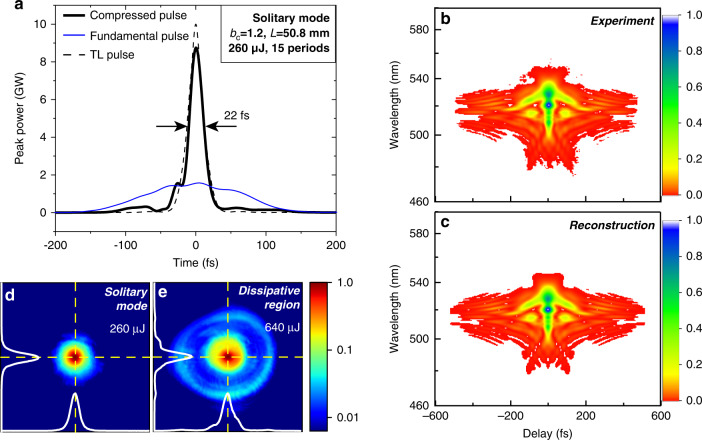
High-efficiency pulse compression under the solitary modes. **a** The temporal intensity profiles of the compressed pulse (solid black) and the incident pulse (solid blue). The dashed black line is the TL pulse. **b**, **c** The measured and reconstructed FROG traces. **d** The spatial mode of the output beam at resonance (*E*_in_ = 260 µJ). The intensity is plotted on a log-scale to highlight the conical emission. The white lines display the *x* and *y* transverse profiles of the beam. **e** Same as (**d**). but with *E*_in_ = 640 µJ in the dissipative region

In the regions *b* > *b*_c_, the space-time coupling breaks the solitary modes and strongly influences the spectral, spatial and temporal profiles. First, we find that the bandwidth ceases to increase almost immediately when *b* > *b*_c_, as shown in Fig. 2a, b. Correspondingly, we observe temporal pulse splitting and asymmetric pulse profiles (Fig. 2c), accompanied by the strong enhancement of conical emission (see Fig. 3d, e below). These correlated effects can be understood as the result of cavity-mode selection in space for different pulse intensities over time (Fig. 1a). When the pulse peak intensity is higher than the critical value, the temporal centre mismatches the cavity resonance and experiences fast divergence due to the strong Kerr lensing, which leads to the splitting of pulses in time and strong conical emission (Fig. 1a). Hence, our results directly demonstrate that the optical loss due to conical emission^21,23,24^ can be substantially suppressed by controlling the propagation of femtosecond pulses into the solitary modes. The saturation of the spectral bandwidth, on the other hand, is caused by the SPM process on a temporally split and positively chirped pulse^36^. These correlated effects can be observed for different resonator lengths, indicating their universality. Moreover, they can be well reproduced by the 2D NLSE simulations with an accuracy of *E*_in_ within 10% of the experimental values (see SM). We further note that even under the solitary modes, the pulse temporal profiles still change, though this change is not as significant as that in the dissipative region. As a result, the solitary modes here are quasi-stationary in nature and eventually cease to exist after propagating through ~20 layers of Kerr media due to the energy loss, material dispersion and nonlinear phases imposed by SPM.

In Fig. 2d, e, we plot the time-frequency analysis of the output pulses under different conditions (see SM). Under solitary propagation, we find that the majority of the optical energy is linearly chirped (Fig. 2d), while a significant amount of nonlinear chirp can be observed in the long-wavelength region in the dissipative region (Fig. 2e). Since the pulses passed through the same amount of Kerr media in both cases, we can exclude that this difference is caused by the higher-order dispersion of materials or carried by the input pulses. On the other hand, when the femtosecond pulses are split and asymmetric in time, a nonlinear frequency chirp can be produced by space-time coupling and the SPM process^37^. Indeed, as we have shown in Fig. 1d, complex pulse profiles have already appeared after a few material layers in the dissipative region. The higher-order dispersion here has important impacts on the pulse compression. Even with appropriate compensation of negative GDD, strong pedestals spanning ~100 fs in time can still be clearly observed for the compressed pulses, which contributes to a temporal energy loss of ~24% (see SM). In contrast, under the solitary modes, the temporal energy loss can be suppressed to <10%, which allows clean pulse compression to nearly the Fourier limit (see Fig. 3a).

### High-efficiency pulse compression with the solitary modes

The generation of gigawatt, temporally confined spatial solitons in this work can significantly improve the SCG and pulse compression in condensed media. Here, we summarize three advantages. First, as evidenced in Fig. 1c and d, the solitary modes can maintain high intensity in both space and time, supporting sustainable nonlinear light-matter interactions over many layers of Kerr media. As an example, we send 260 μJ, 170 fs pulses through a 15-layer PLKM with *L* = 50.8 mm under the resonant condition. The spectrum of the output pulse is significantly broadened, corresponding to a TL pulse duration of 22 fs. The chirp of the output pulses is then compensated by a set of chirped mirrors (PC1611, Ultrafast Innovations), which supplies a total negative GDD of −1200 fs^2^ over 850–1200 nm. The duration of compressed pulses from this single-stage compressor is close to the TL pulse, as shown in Fig. 3a. The total transmission is ~85%, which includes an ~10% loss from the reflections on the material layers and an ~5% loss from the collimation optics and chirped mirrors. The experimental and reconstructed FROG traces of the compressed pulses are plotted in Fig. 3b, c, respectively. Remarkably, the single-stage SCG bandwidth of our result is ~50% broader than the previous multiplate SCG under similar conditions^24^. Second, the single-peaked temporal profile under solitary modes (Fig. 1d) also avoids the generation of higher-order dispersion, which circumvents the usage of custom-designed chirped mirrors or pulse shapers^23,24^. This is evidenced by the fact that clean pulse compression (Fig. 3a) is achieved by compensating only for the second-order dispersion. Last but foremost, the spatial loss induced by conical emission is strongly suppressed with solitary propagation. As shown in Fig. 3d, the conical radiation contributes only <10% of the total output energy when the propagation is on resonance, and this contribution can increase to ~35% in the dissipative region (Fig. 3e). Overall, by combining the broad SCG spectrum and the suppression of the losses in space and time, we achieve a fivefold increase in the peak power from a single-stage compressor (Fig. 3a). This result is among the largest increases in pulse peak power from single-stage compressors with condensed media (see SM).

**Fig. 4:**
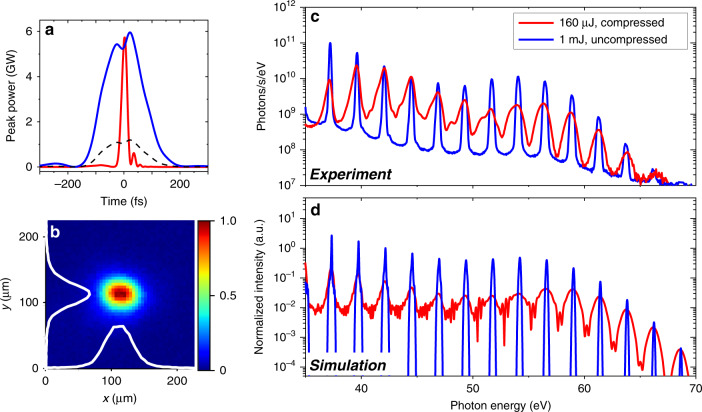
Generation of bright HHG with the compressed pulses. **a** The temporal profiles and peak power of the 160 µJ, compressed (red) and 1 mJ, uncompressed (blue) pulses. The dashed line represents the profile of the 160 µJ, uncompressed pulses for comparison. **b** The spatial profile at the focus of the laser beam of the 160 µJ, compressed pulses. The white lines display the *x* and *y* transverse profiles of the beam. **c** Experimentally measured HHG spectra generated in argon by the 160 µJ, 50 kHz compressed pulses (red) and 1 mJ, 10 kHz uncompressed pulses under the same focusing conditions, reaching a field intensity of ~1.4 × 10^14^ W/cm^2^. **d** Simulation results of the HHG spectra under the same conditions as in (**c**)

The increase in pulse peak power and intensity is especially important for applications in strong-field physics, for example, HHG^38–40^. To demonstrate this, we perform HHG experiments in argon (see “Methods”) driven by compressed and uncompressed (170 fs in the full-width-at-half-maximum (FWHM)) pulses for comparison. By controlling the propagation into the solitary modes, we achieve high-efficiency compression of 200 μJ pulses down to ~24 fs in the FWHM at a repetition rate of 50 kHz. The compression efficiency after the chirped mirrors is ~85%, and an additional loss of ~5% is induced by multiple reflections on the silver mirrors and the vacuum window, leaving pulse energy of ~160 μJ for HHG. The peak power is ~6 GW, which is the same as that of the 1 mJ, uncompressed pulses (Fig. 4a). The repetition rate for the 1 mJ, the uncompressed pulse is 10 kHz, leading to an average power similar to that of the compressed situation. In both cases, the focal spot diameter at the gas cell is ~100 μm (1/e^2^ intensity). The spatial profile at the focus for a 160 μJ, the compressed pulse is shown in Fig. 4b, which exhibits a high-quality Gaussian profile without spatial filtering. Here, we intentionally limit the laser intensity for HHG to ~1.4 × 10^14^ W/cm^2^ to avoid early ground-state depletion on the front edge of the driving pulses^41–43^. The ionization probabilities at the pulse temporal centre are estimated to be 2.5% and 12% for the compressed and uncompressed pulses, respectively (see SM). Under this condition, the cut-off energy is determined by *hv*_*c*_ = *I*_*p*_ + 3.17*U*_*p*_^44–46^, where *I*_*p*_ is the ionization potential of the atom and *U*_*p*_ ≈ *I*_*L*_*λ*^2^ is the ponderomotive energy of an electron in a laser field of intensity *I*_*L*_. The experimentally measured HHG spectra are shown in Fig. 4c. The HHG source shows long-term stability in the photon flux (see SM) when no pointing-stabilization setups are installed, highlighting that the pulse compression is simple (no vacuum-gas interfaces), reliable and stable. As shown in Fig. 4c, the efficient pulse compression of the 160 μJ pulses can extend the cut-off energy until it is equivalent to that generated by the 1 mJ, uncompressed pulses. Notably, in this energy range, the HHG intensity driven by the 160 μJ, uncompressed pulse is so low that effective measurement becomes impossible. This result unambiguously demonstrates the high spatiotemporal quality of the compressed pulses, excluding the strong spatial chirp and nonlinear effects previously reported for SCG in condensed media^47^.

As shown in Fig. 4c, the line-width of harmonic orders driven by 1 mJ, uncompressed pulses is narrower than that of harmonic orders driven by 160 μJ, compressed pulses, which is a result of the interference of HHG emission from multiple optical cycles of the driving pulses^43^. This leads to a higher peak brightness of the harmonic orders in the former condition, while the overall HHG fluxes are similar in both cases. These observations can be well reproduced by our numerical simulations, which take both the single-atom response and propagation effects into account (Fig. 4d, see “Methods”). Furthermore, when the spot size at the focus is further reduced, hence increasing the peak intensity, the HHG cut-off energy can be further increased when driven by compressed pulses, which is not possible for long pulses^43,48^. This phenomenon highlights the advantage of using short-duration laser pulses for HHG, which can greatly reduce the ionization process and limit the ionization probability in the phase-matching regime. Moreover, the reshaping and depletion of the laser pulse can be suppressed due to the decrease in the free-electron density.

### High-efficiency mode self-cleaning under resonant conditions

**Fig. 5:**
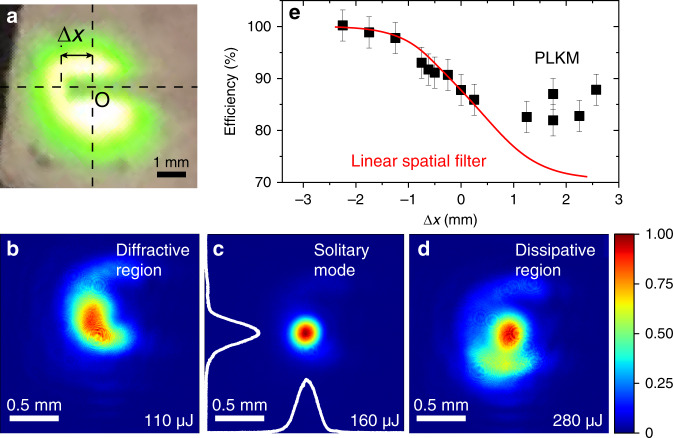
High-efficiency mode self-cleaning under the resonant conditions. **a** Camera picture of the modulated laser beam after the beam blocker for Δ*x* ≈ 1.25 mm. O is the centre of the beam profile, and Δ*x* is the transverse blocker position. **b**–**d** The output beam profiles measured in the far field for the spatially modulated input beam shown in (**a**). in the diffractive region, at the solitary mode and in the dissipative region, respectively. The lines in (**c**). represent the horizontal and vertical beam profiles in the solitary mode. **e** The filtering efficiency as a function of the blocker position (Δ*x*) for the PLKM resonator and a linear spatial filter

Finally, we report the high-efficiency self-cleaning of spatial modes enabled by solitary beam propagation in the PLKM resonators. The improvement in the output spatial mode, in terms of the circularity and the intensity profile, can already be observed after the fundamental laser beam propagates through the PLKM (see SM). To further investigate the mode self-cleaning effect, we introduce substantial perturbance to the beam profile by inserting a cylindrical beam blocker with a diameter of 0.8 mm into the laser beam, which has an FWHM size of ~3 mm (Fig. 5a). The spatially modulated laser beam is then focused into a resonator with *b*_c_ = 0.5 and *L* = 25.4 mm, consisting of 20 layers of Kerr media. As shown in Fig. 5b–d, when matched with the cavity resonance, the output mode is significantly cleaned and transforms into the solitary modes. In Fig. 5e, we plot the filtering efficiency as a function of the blocker transverse position Δ*x*, with a larger Δ*x* inducing greater spatial modulation (Fig. 5a). The PLKM resonator can support a high filtering efficiency (>85%) across a large range of spatial modulations, in direct contrast to an ideal linear spatial filter (see SM). This result suggests that the solitary modes here represent attractors of the (2 + 1)D cubic NLSE^49^. There must be an efficient pathway in which the laser energy in the higher-order spatial modes can be transferred to the solitary modes through repetitive Kerr interactions. This is consistent with the spatial self-organization of laser beams, previously observed under nonlinear interactions in filamentation^50^, self-focusing collapse^51^ and multimode fibres^52,53^.

## Discussion

In this work, we demonstrated the formation of the solitary modes under pulse energies ranging from 100 to 600 μJ, which can be achieved by simply changing the resonator length *L*. This demonstration illustrates that our method is highly flexible and energy/power scalable. In the following, we consider the general scaling rule of our approach. As shown in Fig. 1b, to a good approximation, the resonant conditions can be expressed as $\frac{{w^2}}{{\lambda L}} = \frac{{0.81}}{{b_c}}$. As a result, the overall dimension of the cascade resonator is limited by ${\it{L}}_{{\mathrm{CR}}} \, > \, 2.46\frac{{E_{{\mathrm{in}}}\phi _m}}{{\pi \lambda \Delta \tau I_d}}$ (see SM), where *ϕ*_*m*_ is the total nonlinear phase and *I*_*d*_ is the damage intensity threshold (*I*_*d*_) of the Kerr media. First, this scaling law is universal and does not depend on the details of the Kerr media, such as the thickness or nonlinear coefficients. Second, the overall dimension of the cascade resonator linearly scales with the pulse energy *E*_in_. More interestingly, the scaling rule is similar to that of HCFs, i.e., ${\it{L}}_{{\mathrm{HCF}}} \, > \, \frac{{E_{{\mathrm{in}}}\phi _m}}{{\lambda \Delta \tau I_{{\mathrm{th}}}}}$^54,55^, while it is the ionization intensity threshold (*I*_th_) of the gas media that limits the length of the HCF (see SM). Given that the *I*_th_ of gases is generally much higher than the *I*_*d*_ of solids, the scaling rule shows that our method requires longer dimensions than those of the HCF technique. Nevertheless, the free-space geometry of our method allows us to fold the beam paths, which could lead to compact SCG and pulse compression devices. In fact, similar ideas have recently been investigated for all-solid multi-pass cells^56,57^, although the possibility of generating solitary states has not been investigated. Finally, for pulse compression to ~20 fs, our method exhibits good spectral homogeneity (~95%) across the beam profile (see SM). However, the Kerr lenses here are wavelength-dependent, and the resonant condition cannot be preserved for all wavelengths when the spectral bandwidth is very broad. As a result, we expect high-efficiency soliton formation and high spectral homogeneity to be challenging for the compression of few-cycle pulses.

In summary, we experimentally investigate the spatiotemporal propagation of strong femtosecond pulses in PLKM resonators and reveal its influence on the stability of optical solitons. Taking advantage of the unique characteristics of these solitary modes, we demonstrate high-efficiency SCG, pulse compression and spatial mode self-cleaning. We believe that our method can provide a highly efficient, simple, stable and flexible solution for SCG and pulse compression, especially for state-of-the-art Yb-based lasers. These results are also relevant to a wide range of applications, such as Kerr-lens mode locking^58^, ultrashort-pulse generation^21,23,24^ and high-energy wavelength scaling^22^. Moreover, the results here reveal the general features of the space-time coupling of solitons under periodically modulated Kerr nonlinearity, which may reinforce the theory and improve our understanding of soliton formation under similar periodic “potentials” in many other fields of nonlinear optics, including waveguide arrays^59,60^, periodic refractive-index gratings^61,62^ and photonic crystal fibres^63^, as well as in condensed matter physics^64,65^ and in biology^66^.

## Methods

### Experimental setup

For our experimental studies on the PLKM resonators, we employed a Yb:KGW amplifier laser with a pulse duration of 170 fs at *λ* = 1030 nm. TL femtosecond pulses with p polarization were focused to a beam waist of 140 μm. A half-waveplate and polarizer combination was installed to continuously adjust the incident pulse energy up to 1 mJ. The PLKM was composed of polycrystalline Al_2_O_3_ thin plates as the Kerr medium, which was placed at the Brewster angle to minimize the reflection loss. The average reflection loss was suppressed to <0.5%. In the experiments, we investigated resonators with four different cavity lengths: *L* = 25.4, 50.8, 76.2, and 101.6 mm. We implemented 20 periods for the resonator with *L* = 25.4 mm, whereas the number of periods was reduced to ~10 for the other resonator lengths due to the limited laboratory space. For the SCG and pulse compression experiments, we implemented 15 periods for *L* = 50.8 mm.

### Numerical simulation with the NLSE

The NLSE for forward propagation with radial symmetry is given by^67^

2 $$\begin{array}{l}\frac{{\partial U}}{{\partial z}} = \frac{j}{{2n_0k_0}}T^{ - 1}\nabla _ \bot ^2U + jDU + \\ j\frac{{\omega _0}}{c}n_2T\left[ {\left( {1 - \chi _K} \right)\left| U \right|^2 + \chi _K\displaystyle\mathop{\int}\nolimits_{-\infty}^{t} {h\left( {t - t^{\prime}} \right)\left| {U\left( {t^{\prime}} \right)} \right|^2{\mathrm{d}}t^{\prime}} } \right]U\end{array}$$

Here *t* is the retarded time *t*−*z*/*v*_*g*_, with *v*_*g*_ being the group velocity near the carrier frequency *ω*_0_, *k*_0_ being the wave vector in a vacuum, $D = \frac{{k^{\prime\prime}}}{2}\left( {j\partial _t} \right)^2$representing the dispersion term, $T = \left( {1 + \frac{{j\partial _t}}{{\omega _0}}} \right)$, *χ*_*K*_ being the coefficient for the Raman response and $h\left( t \right) = \frac{2}{3}\frac{{\left( {\tau _1^2 + \tau _2^2} \right)}}{{\tau _1\tau _2^2}}e^{ - t/\tau _2}\sin \left( {t/\tau _1} \right)$ being the Raman response function. The split-step method was used to numerically solve the NLSE. The numerical error in each step was carefully kept at O(d*z*^3^), where d*z* is the step size of the propagation. The parameters used in the NLSE simulation are listed in Table S1 of the SM.

### Time-frequency analysis

The time-frequency analysis was carried out using the Wigner-Ville distribution^68^, which is given by

3 $$W\left( {t,\omega } \right) = \frac{1}{{2\pi }}{\int} {E^ \ast \left( {t - \frac{1}{2}\tau } \right)e^{ - j\tau \omega }E\left( {t + \frac{1}{2}\tau } \right){\mathrm{d}}\tau }$$

Here, the electric fields of the femtosecond pulses are obtained by

4 $$E\left( t \right) = \frac{1}{{\sqrt {2\pi } }}{\int}_{\! - \infty }^\infty {\sqrt {I\left( \omega \right)} e^{ - j\varphi \left( \omega \right)}e^{ - j\omega t}{\mathrm{d}}\omega }$$

where *I*(*ω*) and *φ*(*ω*) are the spectral intensity and phase directly measured with FROG. The analysis satisfies the marginals, that is, ${\int} {W\left( {t,\omega } \right){\mathrm{d}}\omega } = I\left( t \right)$ and ${\int} {W\left( {t,\omega } \right){\mathrm{d}}t} = I\left( \omega \right)$.

### HHG setup

To initiate the HHG process, the laser beams were recollimated and focused onto a cylindrical gas cell with a focal spot diameter of ~90 μm. We note that the beam after the PLKM compressor could be well focused to a Gaussian profile, even though no spatial filtering was applied here. The gas cell, which had an inner diameter of 1.5 mm, was supplied with argon gas and positioned near the focus of the laser beam to optimize the phase matching for the short trajectories^69^. The HHG spectrum was measured by an EUV spectrometer after blocking the driving optical beam with a 200 nm aluminium film, while the brightness of the HHG was characterized by a calibrated diode, as well as the response function of the spectrometer.

### HHG simulation

Since the high harmonics were coherently generated from all atoms in the gas medium, two parts were considered to fully describe the experimentally measured HHG spectra: (i) the single-atom response, i.e., the dipole induced by the driving laser field, which can be calculated by solving the time-dependent Schrödinger equation (TDSE) or by an equivalent simpler model; and (ii) the macroscopic response, which can be obtained by solving the three-dimensional Maxwell wave equations for both the fundamental driving laser and the high-harmonic fields, as described in detail in ref. ^70^. In this work, the solution of the TDSE was replaced by using a quantitative rescattering (QRS) model^71,72^. For the propagation of a driving laser pulse in a gas medium, we included the effects of dispersion, Kerr nonlinearity, and plasma; therefore, spatiotemporal reshaping of the laser pulse may occur. In the simulations, we chose to set the parameters as close as possible to those in the experiment. The laser wavelength was 1030 nm. For uncompressed and compressed pulses, we used pulse energies of 1 mJ and 150 μJ and pulse durations of 170 and 25 fs, respectively, with an accuracy of 10% compared to the experiments. The beam waist in both cases was 50 μm, reaching the same laser intensity of 1.4 × 10^14^ W/cm^2^ at the focus. Gaussian temporal and spatial beam profiles were assumed. The gas jet was modelled to occur after the laser focus with an effective length of 1.5 mm and a uniform density distribution. The gas pressure was adjusted to achieve the best agreement with the experimental observations.

## Supplementary information

Supplementary Information for Solitary beam propagation in periodic layered Kerr media enables high-efficiency pulse compression and mode self-cleaning
