# Supplementary material for: Solitary beam propagation in periodic layered Kerr media enables high-efficiency pulse compression and mode self-cleaning
Source: Light Sci Appl. 2021 Mar 10;10:53. doi: 10.1038/s41377-021-00495-9 (PMC7946960; doi:10.1038/s41377-021-00495-9)
Supplement: Supplementary file 1 — Supplementary Information for Solitary beam propagation in periodic layered Kerr media enables high-efficiency pulse compression and mode self-cleaning [file 41377_2021_495_MOESM1_ESM.docx]

**Supplementary Information for**

**Solitary beam propagation in periodic layered Kerr media enables high-efficiency pulse compression and mode self-cleaning**

Sheng Zhang^1†^, Zongyuan Fu^1†^, Bingbing Zhu^1†^, Guangyu Fan^2^, Yudong Chen^1^, Shunjia Wang^1^, Yaxin Liu^1^, Andrius Baltuska^2^, Cheng Jin^3^, Chuanshan Tian^1^, Zhensheng Tao^1^

^1^State Key Laboratory of Surface Physics and Department of Physics, Fudan University, Shanghai, China

**^2^**Institute of Photonics, TU Wien, Gusshausstrasse 27/387, Vienna, Austria

***^3^*** *Department of Applied Physics, Nanjing University of Science and Technology, Nanjing, Jiangsu 210094, China*

^†^These authors contributed equally to this work.

*Corresponding authors: Dr. Zhensheng Tao, ZhenshengTao@fudan.edu.cn.

**This file includes:**

a) Supplementary Text

b) Figs. S1 to S16

c) Table S1 and S2

1. **Schematics of the experimental setup**

The schematics of the experimental setup is shown in Fig. S1. For our experimental studies on the PLKM resonators, we employed a Yb:KGW amplifier laser with a pulse duration of 170 fs at *λ*=1030 nm. Transform-limited femtosecond pulses with p polarization are focused to a beam waist of 140 μm, with a half-waveplate and polarizer combination installed for adjusting the incident pulse energy up to 1mJ continuously. The PLKM is composed of polycrystalline Al_2_O_3_ thin plates as the Kerr medium, placed at the Brewster angle to minimize the reflection loss. The reflection loss is suppressed to <0.5%. The nominal thickness of the plates is fixed to be 0.4 mm, except that the thickness of the first plate is half of this value to provide the correct initial spatial phase. The input surface of the PLKM is placed at the beam focus, with the distance between the neighboring plates equal to the resonator length *L*. In the experiments, we investigated resonators with 4 different lengths: *L*=25.4, 50.8, 76.2, 101.6 mm. We implemented 20 periods for the resonator with *L*=25.4 mm, whereas the number of periods reduces to ~10 for the other resonator lengths, due to the limit of laboratory space. For the supercontinuum generation (SCG) and pulse compression experiments, we implemented 15 periods for *L*=50.8 mm.

**
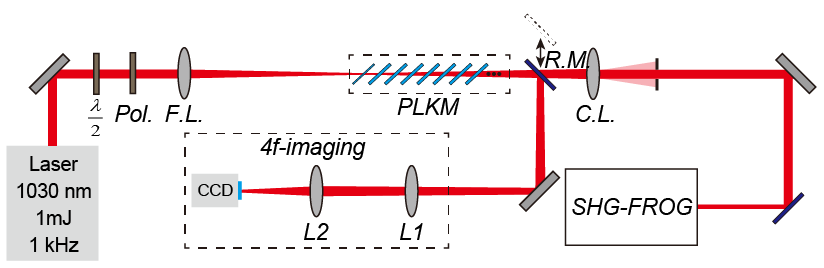
**

**Figure S1.** Schematic of the experimental setup. Pol., polarizer; F.L., focusing lens; R.M., retrievable mirror; C.L., collimating lens; L1 and L2, lenses for 4f-imaging.

1. **Effective nonlinear and dispersive thickness of the Kerr media**

Because of the Brewster angle ($\theta_{B}$) implemented in our experiments, the thickness of the layers of the Kerr media needs to be re-evaluated for the nonlinear and dispersive interactions, respectively. The correction on the thickness is attributed to the refraction of light on the air-medium interface, as shown in Fig. S2. The nominal thickness is *l*. Considering the refraction of light, we have $n_{0}\sin\theta=\sin\theta_{B}$. Here we ignore the laser-induced change of the refraction index, which is orders of magnitude smaller. As a result, for the dispersive interaction, the effective length is $l_{eff}^{D}=\frac{l}{\cos\theta}$. For the nonlinear interaction (the Kerr effect), the nonlinear phase is given by $b=\frac{2\pi}{\lambda}n_{2}I_{0}l_{eff}^{N}$. Here, in addition to the increased optical path in the medium ($l_{eff}^{D}$), the transverse beam waist is also increased to $w'=\frac{w}{\cos\theta_{B}}\cos\theta$, leading to a reduced beam intensity. Effectively, the nonlinear length is $l_{eff}^{N}=\frac{l\cos\theta_{B}}{\cos^{2}\theta}$, assuming a normal incidence of the laser beam. Given that $\theta_{B}=60.5^{\text{o}}$, $n_{0}=1.75$ for Al_2_O_3_ at $\lambda=1030 \text{nm}$ and l = 400 μm, we get $l_{eff}^{N}\approx260 \text{μm}$.


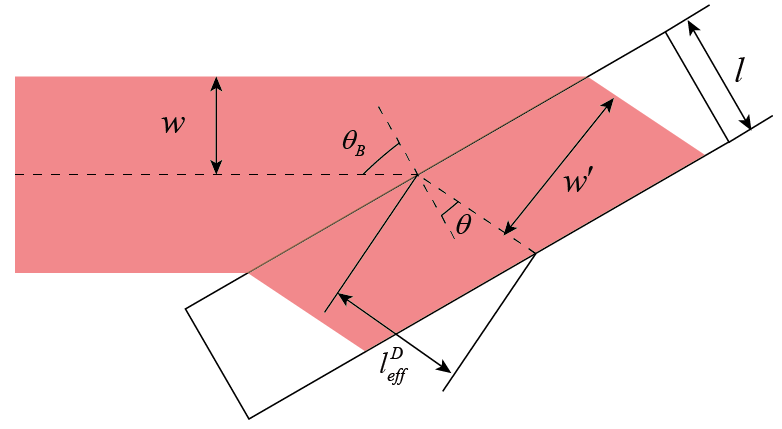


**Figure S2.** Illustration of light refraction at the air-medium interface.

1. **Experimental determination of *b*_c_**

The solitary modes are identified experimentally by monitoring the far-field beam size and the quality of the spatial modes as a function of the incident pulse energy under a specific resonator length *L*. In Fig. S3 (b-e), we summarized the variation of the far-field beam size as a function of the incident pulse energy and nonlinear phase for four different *L*s. The solitary mode is determined when the beam size reaches its minimum value. We found that the quasi-stable oscillatory region is generally narrower than the prediction of the FKD model (dashed lines), which can be attributed to the space-time coupling and temporal pulse splitting above the resonance.

**
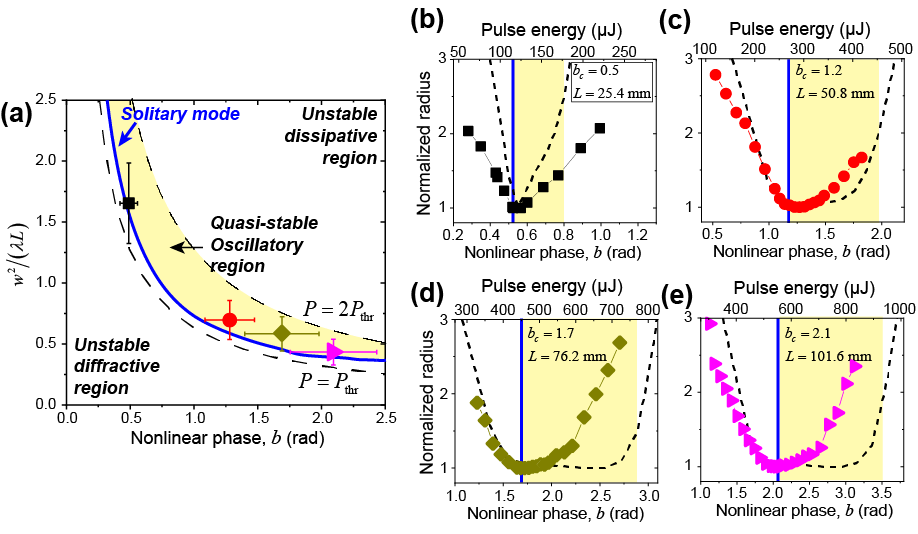
**

**Figure S3.** (a) Universal relationship of the normalized beam radius squared *w*^2^/*λL* as a function of the nonlinear phase b for the resonator solitary modes. Same as Fig. 1(b). (b-e) Variation of the normalized far-field beam radius as a function of nonlinear phase and pulse energy for the corresponding *b*_c_ and *L* in (a). The FKD simulation results for the beam radius are represented by the dashed lines. For *b*_c_=0.5, *L*=25.4 mm, we simulate the propagation through 20 layers of the Kerr medium, while, for the other cases, 10 layers are implemented.

Here, the critical nonlinear phase of a resonant mode is defined by $b_{c}=\frac{2\pi}{\lambda}n_{2}l\left(\frac{2E_{c}}{t_{p}\pi w^{2}}\right)$, where *E_c_* is the pulse energy under the cavity resonance, *t_p_* the pulse duration and *w* the beam radius. Experimentally, we measure the beam radius and pulse duration at each layer of the media using the 4-f imaging and SHG-FROG setups, respectively. The averaged values of *w* and *t_p_* are used to determined the strength of *b_c_* in each case. In Fig. S4, we plot the variations of beam radius and pulse duration on each plate measured using 4-*f* imaging and SHG-FROG setups for *b_c_*=0.5, *L*=25.4 mm and *b_c_*=1.0, *L*=50.8 mm, respectively. The standard deviation of the data contributes to the error bars in Fig. 1(b).


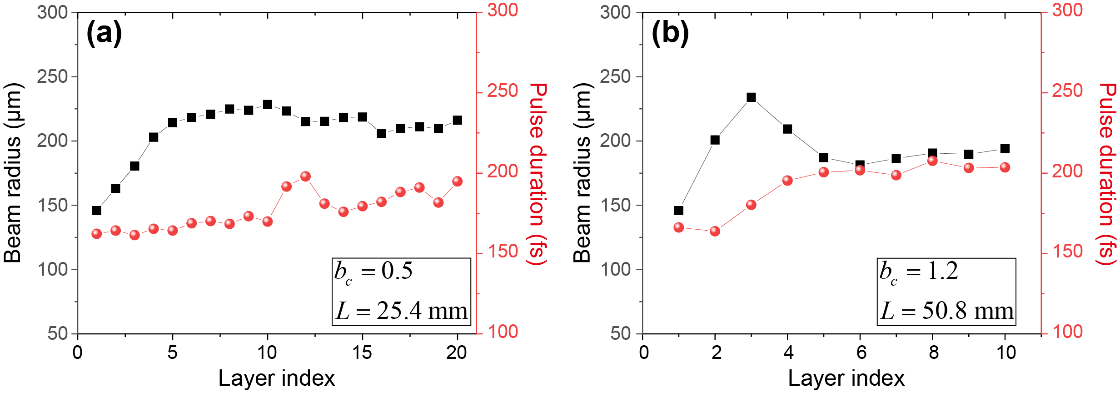


**Figure S4.** (a) The experimentally measured beam radius and pulse duration on each layer of the Kerr media under the critical nonlinear phase *b_c_*=0.5, *L*=25.4 mm. (b) Same as (a) for *b_c_*=1.2, *L*=50.8 mm

The relationship between $w^{2}/\lambda L$ and the nonlinear phase *b* for the beam with a threshold power *P_thr_* can be derived as the following. Because $P_{thr}=P_{cr}\gamma n_{0}$, we have $P_{thr}=\frac{\lambda^{2}}{2\pi n_{2}}\frac{L}{l}$. The nonlinear phase applied by the laser beam when its power is *P_thr_* is then given by $b_{thr}=\frac{2\pi}{\lambda}n_{2}l\frac{2P_{thr}}{\pi w^{2}}=\frac{2}{\pi}\frac{\lambda L}{w^{2}}$. As a result, we obtain $\frac{w^{2}}{\lambda L}=\frac{2}{\pi}\frac{1}{b}$ for the curve of *P* = *P_thr_* in Fig. 1(b).

1. **Beam propagation in the quasi-stable oscillatory region**

According to the FKD model, we find that there exists a quasi-stable oscillatory region for beam propagation in the PLKM when the nonlinear phase applied by the incident beam, *b*, is greater than the critical nonlinear phase *b_c_* and pulse power is less than 2*P*_thr_. In Figs. S5(b) and (c), we plot the variations of the beam size obtained from our FKD model as the beam passes for *b*=1.0, 1.2, 1.5 and 2.0 *b_c_* through the PLKM resonator with two critical phases: *b_c_*=0.5 and 1.0. By increasing the nonlinear phase, the beam size experiences strong oscillation as the beam propagates in the PLKM. The amplitude of oscillation increases, as the nonlinear phase deviates from the critical phase corresponding to the stationary modes. When the nonlinear phase is higher than the upper limit *P*=2*P*_thr_, the beam size grows out of limit in only few periods of the resonator.


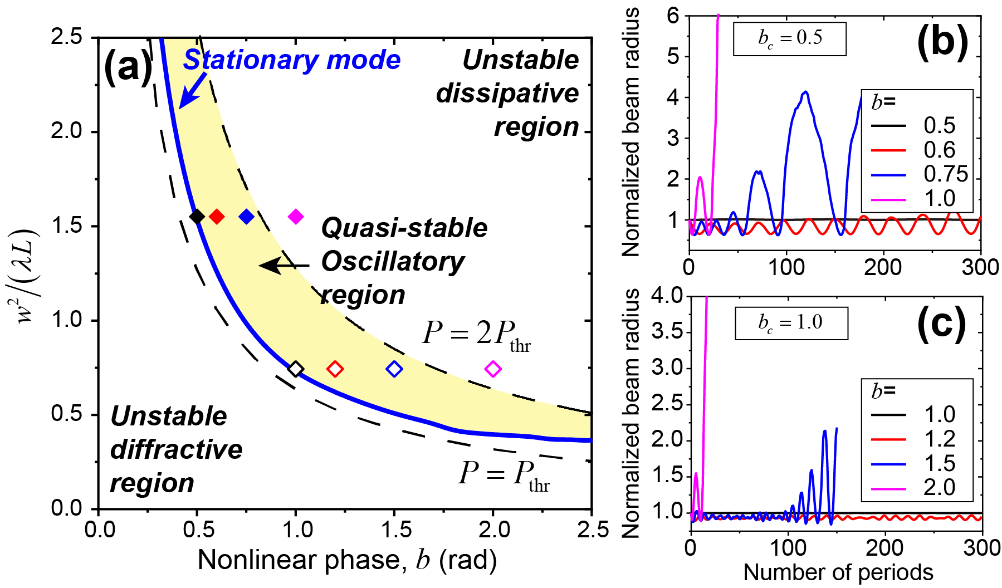


**Figure S5.** (a) The universal relationship of $w^{2}/\lambda L$ and the nonlinear phase *b* for the stationary modes of the PLKM resonators. Different regions for the resonator stabilization are labeled. The quasi-stable oscillatory region is highlighted by the yellow color. (b) and (c) The model results for the evolution of beam radius in a nonlinear resonator with *b_c_*=0.5 and 1.0, respectively. The lines with different colors show the beam size evolution under the conditions labeled by the diamonds with the same color in (a).

1. **Temporally confined propagation on resonance**

In Fig. 2(f), we present the evolution of pulse temporal profiles as the femtosecond pulses propagate through the PLKM with *L*=50.8 mm on and above the resonance. As shown in Fig. S5, similar temporal localization and pulse splitting can be observed for resonators with *L*=25.4 and 101.6 mm.

**
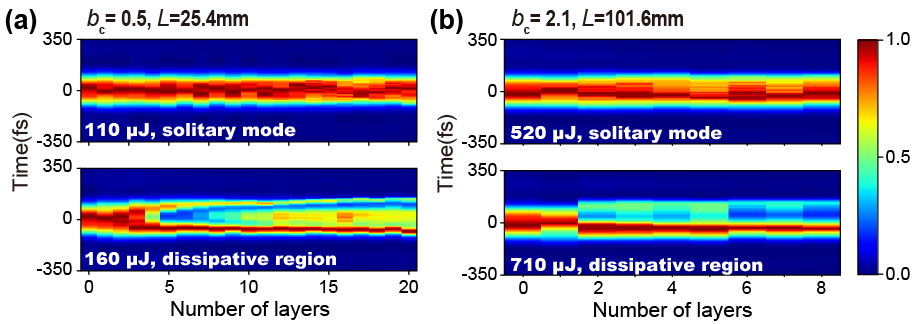
**

**Figure S5.** Evolution of temporal intensity profiles as the pulses propagate through the PLKM on and above the resonance for (a) *b*_c_ = 0.5 , *L* = 25.4 mm and (b) *b*_c_ = 2.1 , *L* = 101.6 mm

1. **Numerical simulation with NLSE**

The NLSE for forward propagation with radial symmetry is given by [S1]

$\frac{\partial U}{\partial z}=\frac{j}{2n_{0}k_{0}}T^{-1}\nabla_{\perp}^{2}U+jDU+j\frac{\omega_{0}}{c}n_{2}T\left[\left(1-\chi_{K}\right)\left|U\right|^{2}+\chi_{K}\int_{-\infty}^{t} h\left(t-t'\right)\left|U\left(t'\right)\right|^{2}\text{d}t'\right]U$. (S1)

Here, *t* is the retarded time $t-z/v_{g}$ with *v_g_* the group velocity near the carrier frequency *ω*_0_, *k*_0_ the wave vector in vacuum, $D=\frac{k''}{2}\left(j\partial_{t}\right)^{2}$represents the dispersion term, operator *T* is given by $T=\left(1+\frac{j\partial_{t}}{\omega_{0}}\right)$,$\chi_{K}$is the coefficient for the Raman response and $h\left(t\right)=\frac{2}{3}\frac{\left(\tau_{1}^{2}+\tau_{2}^{2}\right)}{\tau_{1}\tau_{2}^{2}}e^{-t/\tau_{2}}\sin\left(t/\tau_{1}\right)$ is the Raman response function. The parameters used in the NLSE simulation is listed in Table S1.

The split-step method is used to numerically solve the NLSE. The numerical error in each step is carefully controlled at the level of O(d*z*^3^) where d*z* is the step size of propagation. The parameters used in our simulation are listed in Table S1. As shown in Fig. S6, our NLSE simulation confirms that the beam can sustainably propagate in the PLKM resonator, when the laser beam matches with the cavity resonance.

**Table S1.** Parameters for the NLSE simulation

|  | Al_2_O_3_ | air |
| --- | --- | --- |
| *n*_2_ (m^2^W^-1^) | 3.0x10^-20^ [S2] | 3.0x10^-23^ [S3] |
| *k’’* (fs^2^mm^-1^) | 32.2 [S4] | 0.0162 [S5] |
| $\chi_{K}$ | 0.16 [S6] | 0.5 [S7] |
| *τ*_1_ (fs) | 13.5 [S6] | 62 [S7] |
| *τ*_2_ (fs) | 40 [S6] | 77 [S7] |


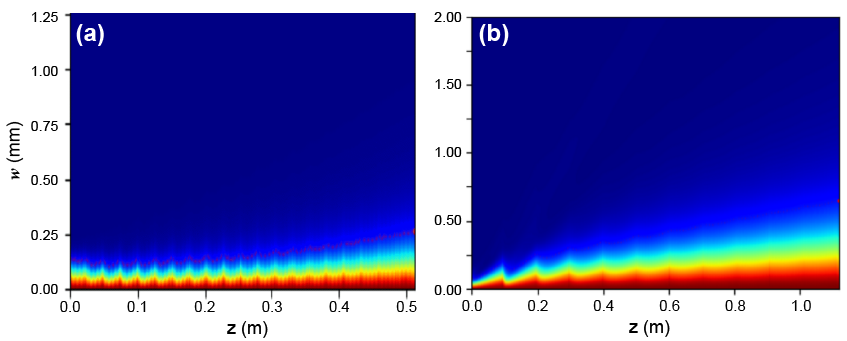


**Figure S6.** (a) The NLSE simulation results of the beam propagation in a PLKM resonator *b*_c_=0.5, *L*=25.4 mm, with the incident beam matching with the cavity resonance. (b) Same as (a) for a PLKM resonator *b*_c_=2.0, *L*=101.6 mm.

1. **Time-frequency analysis**

In Fig. 2(d) and (e), we present the results of time-frequency analysis on the output pulses under the solitary modes and in the dissipative region. The time-frequency analysis is calculated using the Wigner-Ville distribution [S8], which is given by

$W\left(t,\omega\right)=\frac{1}{2\pi}\int E^{*}\left(t-\frac{1}{2}\tau\right)e^{-j\tau\omega}E\left(t\text{+}\frac{1}{2}\tau\right)\text{d}\tau$. (S2)

Here, the electric field of femtosecond pulses are obtained by

, (S3)

where *I*(*ω*) and *φ*(*ω*) are the spectral intensity and phase directly measured with FROG. The analysis satisfies the marginals, that is $\int W\left(t,\omega\right)\text{d}\omega=I\left(t\right)$ and $\int W\left(t,\omega\right)\text{d}t=I\left(\omega\right)$. By applying GDD of -1000 fs^2^, we can compress the pulse in Fig. 2(e), and the resultant Wigner-Ville plot is shown in Fig. S7(a). Correspondingly, the temporal profile of the pulse is given by $I\left(t\right)=\int W\left(t,\omega\right)\text{d}\omega$ and plotted in Fig. S7(b). We observe significant contribution of pedestals spanning ~100 fs in time, as shown in Fig. S7(b). This is in clear distinction from the compression with solitary modes.


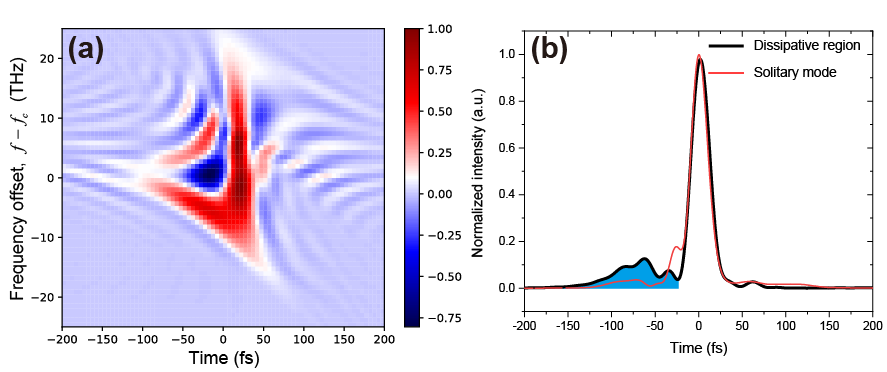


**Figure S7.** (a) Time-frequency analysis of femtosecond pulses of Fig. 2(e) by applying GDD of -1000 fs^2^. (b) Temporal profiles of compressed pulses in (a), in comparison with compressed pulse under the solitary mode.

In Table S2, we summarize and compare the performance of several existing experiments which implemented the multiplate SCG with our results. In this analysis, we extract the temporal profiles of the compressed pulses from Ref.  [S9–S12] and then normalize the intensity to that of the incident pulse, by considering the energy efficiency in the specific experiments. From the comparison, we show that our work represents the highest in energy efficiency, compression ratios and the increment of pulse peak powers among all the single-stage compressors. The 5-time increment of peak power is the best performance among single-stage compressors, and is even comparable to the best performance of a two-stage compressor. This result highlights the great improvement from our method.

**Table S2: Comparison of our work with previous multiplate experiments**

| Ref. | Type | Energy efficiency | Compression ratio of FWHM pulse duration  ($\tau_{in}/\tau_{out}$) | Increment of peak power |
| --- | --- | --- | --- | --- |
| [S9] | Two-stage | 10% | 15.625 | 1.2x |
| [S10] | Two-stage | 25% | 25 | 5x |
| [S11] | Single-stage | 64% | 5.64 | 2.5x |
| [S12] | Single-stage | 75% | 5.55 | 2.4x |
| Our work | Single-stage | 85% | 7.73 | 5x |

1. **Coupled spectral, spatial and temporal evolution of femtosecond pulses**

In our experiments, the spectra of the laser beam were measured in the far field using a spectrometer with an InGaAs detector. We select the on-axis portion of the field to be characterized by the spectrometer and the SHG-FROG. For the spectral measurement, we cut ~10% of the optical energy on axis, while ~30% energy was selected for the time-domain FROG measurement in order to obtain enough signal-to-noise ratio. In Fig. S8(a) and (b), we plot the evolution of the spectrum and temporal profiles as a function of incident pulse energy for the resonator condition: *b_c_*=0.5, *L*=25.4 mm. As shown in Fig. S8(a), the spectral intensity close to the fundamental wavelength significantly increases when the pulse energy is higher than the critical value. Correspondingly, temporal pulse splitting on the axis can be observed, as shown in Fig. S8(b).


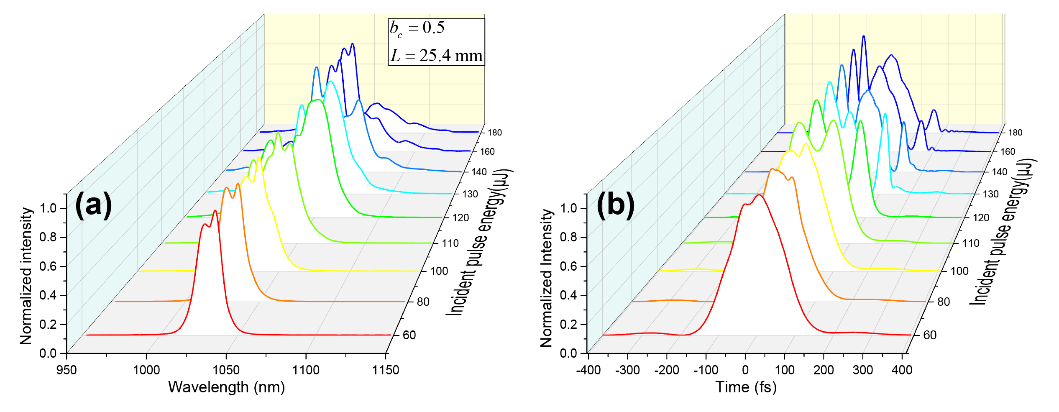


**Figure S8.** (a-b) The experimentally measured on-axis spectral and temporal profiles as a function of the incident pulse energy under the critical nonlinear phase *b_c_*=0.5, *L*=25.4 mm.

The space-time coupled propagation and the corresponding variation spectral, spatial and temporal profiles can be well captured by our 2D NLSE simulation. In Fig. 2(a), we present the quantitative agreement of the NLSE simulation with the experimentally measured beam size and spectral bandwidth for the PLKM resonator with *L*=50.8 mm. The bandwidth here is defined as the spectral width that contains 75.8% of the total energy. In Fig. 2(b-c), we show characteristic variation of the spectral and temporal profiles of the femtosecond pulses below, on and above the resonance. Such agreements for *L*=25.4 and 101.6 mm are shown in Fig. S9 and Fig. S10, respectively, highlighting the universality of this observation.


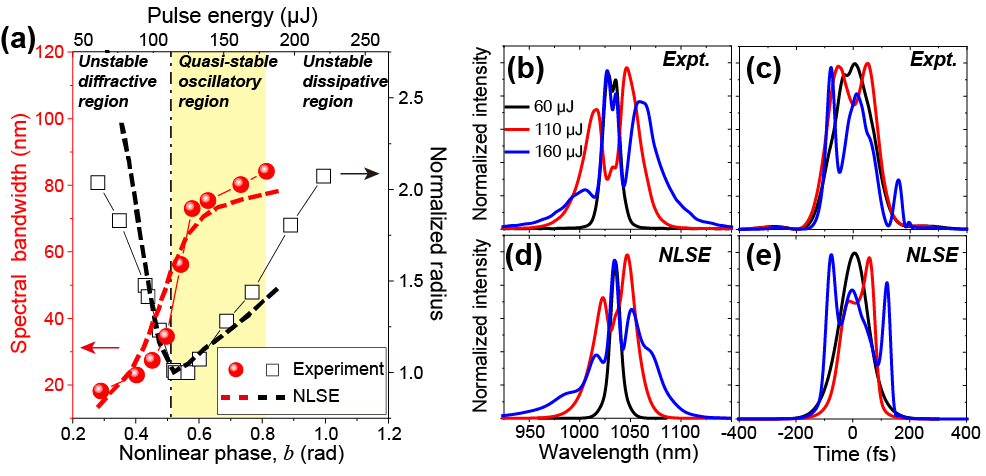


**Figure S9.** (a) The spectral bandwidth and normalized beam radius as a function of the nonlinear phase and pulse energy for a resonator with *b*_c_ = 0.5, *L*=25.4 mm with 20 periods. The dashed lines are the NLSE simulation results. Different regions for the resonator stability are labeled. (b-c) The far-field axial spectral and temporal profiles in different stability regions. (d-e) The NLSE simulation results under the same experimental conditions as in (b-c).


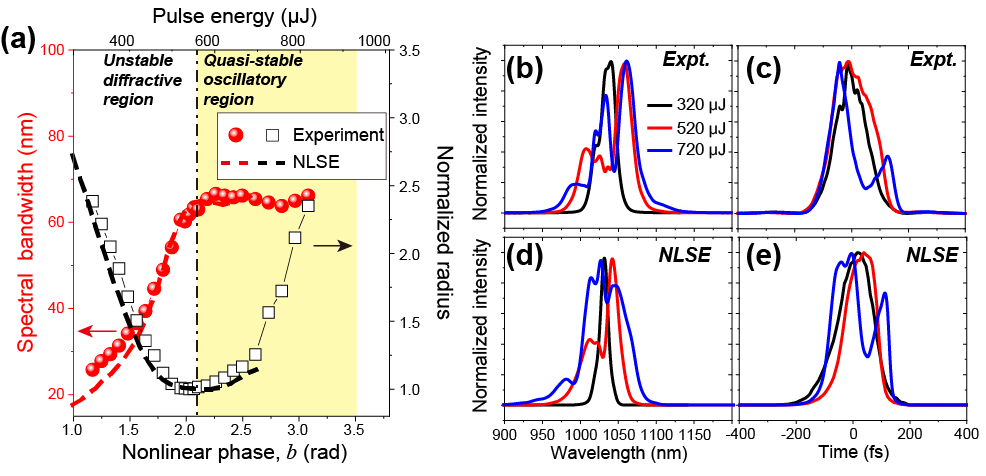


**Figure S10.** (a) The spectral bandwidth and normalized beam radius as a function of the nonlinear phase and pulse energy for a resonator with *b*_c_ = 2.1, *L*=101.6 mm with 8 periods. The dashed lines are the NLSE simulation results. Different regions for the resonator stability are labeled. (b-c) The far-field axial spectral and temporal profiles in different stability regions. (d-e) The NLSE simulation results under the same experimental conditions as in (b-c).

1. **Spatial mode-self-cleaning**

The effect of spatial mode cleaning can already be observed when the fundamental laser beam propagates through the PLKM resonator. In Fig. S11, we plot the variation of spatial profile of the fundamental beam after passing through a resonator with *b_c_*=0.5, *L*=25.4 mm. At the critical nonlinear phase for the best beam-resonator coupling (~110 μJ), the beam profile, including the intensity distribution and roundness, is significantly improved.


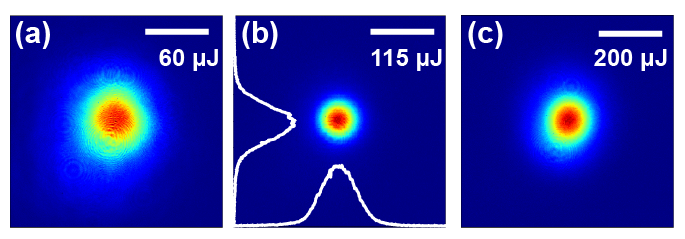


**Figure S11.** The far-field beam profiles measured through a resonator with *b_c_*=0.5, *L*=25.4 mm with the incident pulse energies 60 μJ (a), 115 μJ (b) and 200 μJ (c).

To compare the efficiency of the nonlinear resonator to an ideal linear spatial filter, we implement a numerical simulation to estimate the efficiency of an ideal linear spatial filter under the same profile modulation as in our experiments. This is done by first reproducing the beam profile under the beam blocker in the real space, as shown in Fig. S12(a). Then it is transformed to the reciprocal space through the Fourier transform (Fig. S12(b)). A circular spatial filter with an appropriate size is applied in the Fourier plane to remove the diffracted beam. The size of the spatial filter is adjusted to generate a reasonable single-mode profile as shown in Fig. S12(c), and the largest filter size is used to calculate the filter efficiency. On the other hand, if the filter size is too large, multi-mode beam profile is clearly observed and discarded (Fig. S12(d)).


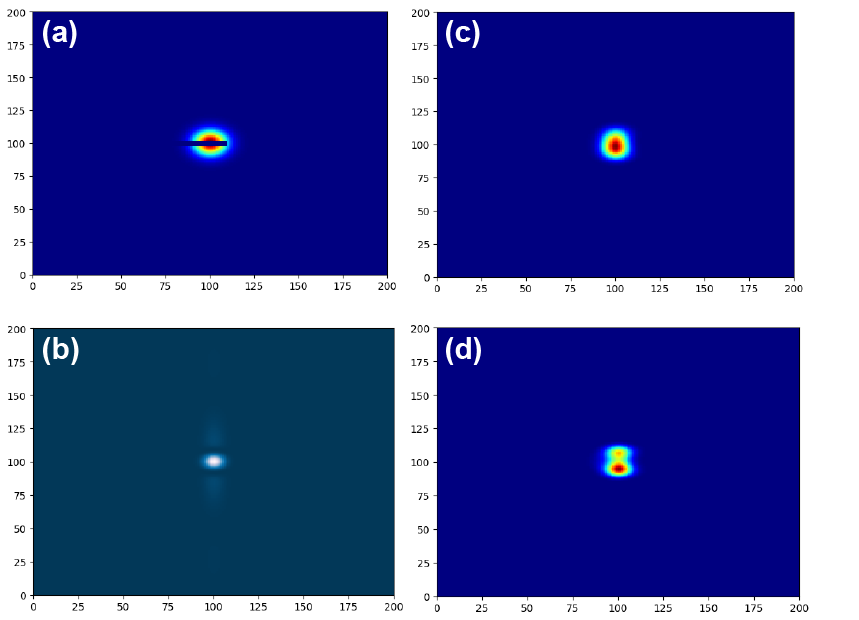


**Figure S12.** (a) The beam profile after the modulation for the modeling of the linear spatial filter. (b) Fourier transform of the pattern in (a). (c) The profile after applying a spatial filter with 10-pixel radius and inverse Fourier transform of the pattern in (b). (d) Same as (c) with a spatial filter of 15-pixel radius.

In the analysis of the experimental data obtained from a nonlinear PLKM resonator, the efficiency of the spatial mode-cleaning is normalized by the transmission efficiency of the PLKM resonator, which allows us to exclude the pure optical reflection loss from the Brewster angles. For each layer of Al_2_O_3_, we can achieve <0.5% reflection loss on average. As a result, this overall reflection loss for a 20-period resonator is ~90%. In addition, under strong spatial modulation of the incident beam, there could exist weak satellite modes beside the main laser mode, as shown in Fig. 4(c). For the calculation of the spatial filtering efficiency, the integral intensity of the satellite beams is excluded, which typically contributes to ~15% of the overall intensity. In Fig. S13, we plot the spatial modes of the input and output beams. The input profiles are recorded by a camera and the output modes are measured using a CCD camera. The efficient spatial self-cleaning can be observed over a wide


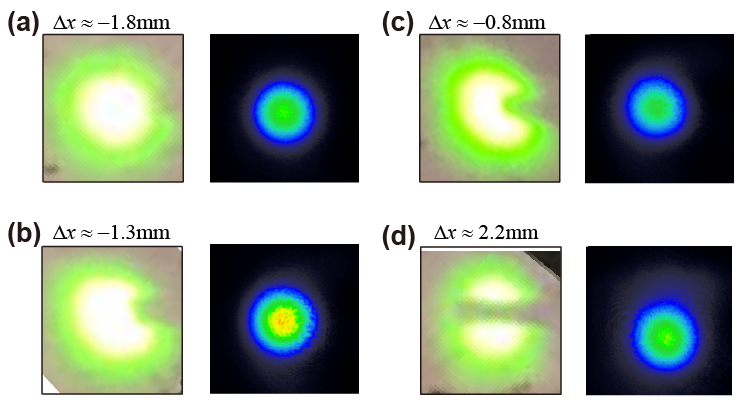


**Figure S13.** Images of the input and output beam modes for different Δ*x* values.

1. **Ionization for uncompressed and compressed laser pulses**

For the uncompressed 170-fs and compressed 25-fs pulse, we can compare with their ionization probabilities with the same peak intensity of 1.44 × 10^14^ Wcm^-2^. The ionization probability with time is calculated by using ADK model [S13], and the laser pulse is assumed as a Gaussian one. The calculated results are shown in Fig. S14. It can be seen that the ionization probability can reach to about 25% (or 12%) at the end of pulse (or at the peak of pulse) for 170-fs pulse, and it is only about 4% (or 2.5%) for 25-fs pulse.

**
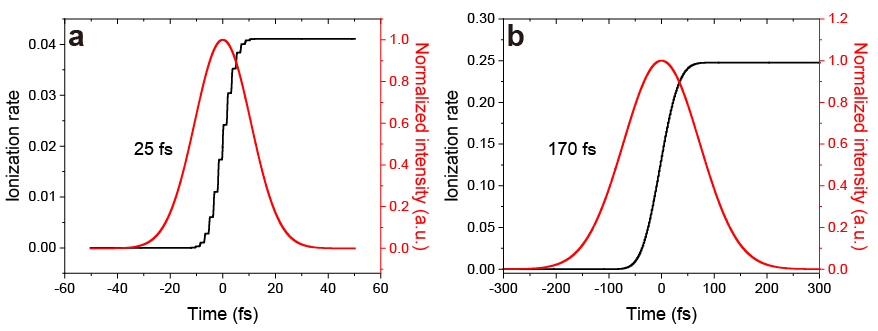
**

**Figure S14.** (a) Ionization rate as a function of time for 25-fs, 1030-nm pulses calculated with the ADK model. (b) same as (a) for 170-fs, 1030-nm pulses.

1. **Stability of the HHG source and the pulse compression system.**

In Fig. S15, we measure the long-term stability of the HHG source driven by the compressed pulses over a period of ~30 min. The overall fluctuation is <2.5% rms, even when the environment temperature varies several degrees. We note that no pointing-stabilization setup was installed for this measurement. The HHG process is a highly nonlinear process, which is sensitive to the energy, pulse durations and spatial profiles of the driving pulses. This result clearly demonstrates the stability and robustness of our method.


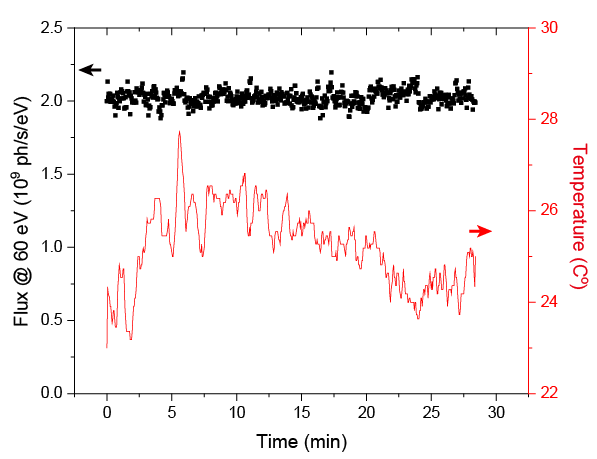


**Figure S15.** Long term stability of the source at 60 eV with a rms deviation of 3%. The measurement of the environment temperature in the same period is plotted as the red solid line.

1. **The scaling rules of the resonator dimension and HCF**

For a spectral broadening factor $F=\Delta\omega/\left(\Delta\omega\right)_{0}$, where Δ*ω* is the spectral bandwidth and (Δ*ω*)_0_ the initial frequency width, the total nonlinear phase required is given by when  [S14]. The nonlinear phase accumulated on each layer is bc, when the propagation of femtosecond pulses is on resonance. As a result, it requires $N=\frac{\varphi_{m}}{b_{c}}$ units of the resonator. As a result, the overall dimension of the PLKM resonator is given by$L_{\text{CR}}\text{=}NL$. To a good approximation, the conditions of the solitary modes (Fig. 1b) can be expressed as$\frac{w^{2}}{\lambda L}=\frac{0.81}{b_{c}}$, and we have the lower limit of the beam waist *w* because of the damage threshold (*I_d_*) of the Kerr media: $w^{2}>\frac{2E_{\text{in}}}{\pi\Delta\tau I_{d}}$. So, in summary, the scaling rule of the PLKM resonator is .

For HCFs, the total nonlinear phase accumulated in the gas medium is given by , where *P*_in_ is the peak power of the incident pulses, *A*_eff_ the effective mode area and $L_{\text{HCF}}$the fibre length. To avoid the self-focusing effect, the peak power *P*_in_ should be lower than the critical power: $P_{in}<P_{cr}=\frac{\lambda^{2}}{2\pi n_{2}}$. As a result, we have . At the same time, the mode area *A*_eff_ is limited by the ionization limit of the gas, which is given by $A_{eff}>\frac{P_{in}}{I_{th}}=\frac{E_{in}}{\Delta\tau I_{th}}$. So, in summary, the scaling rule of the HCFs is given by .

1. **The spectral homogeneity of the compressed pulses over the beam profile.**

To characterize the spatial homogeneity, a spatial filtering experiment is set up under the resonant condition for 1 mJ pulses, and the cavity length *L* is~ 12 cm. The emerging beam is attenuated and sent to far field, yielding a transverse mode radius of ~ 5 mm at a distance of ~2 m. A round aperture with diameter of 1mm is applied to select different portions of the beam along the radius of the transverse mode (see inset of Fig. S16b). The spectrum after the aperture is measured using a spectrometer with an InGaAs detector. The homogeneity of the spectral broadening is characterized following Ref. [S15] by $V\left(r\right)=\frac{\int\left[I\left(\lambda,r\right)I\left(\lambda,0\right)\right]^{\frac{1}{2}}d\lambda}{\int I\left(\lambda,r\right)d\lambda\cdot\int I\left(\lambda,0\right)d\lambda}$, where $I\left(\lambda,r\right)$is the spectral intensity at radial position *r*, and $I\left(\lambda,0\right)$ the on-axis spectral intensity. In Fig. S16a, we plot the experimentally measured spectral distribution, and the homogeneity across the beam profile is shown in Fig. S16b. The intensity-weighted average of the homogeneity is given by $\left\langle V\right\rangle=\frac{\int I\left(r\right)V\left(r\right)rdr}{\int I\left(r\right)rdr}$ , which yields the spectral homogeneity of ~95% across the beam profile.


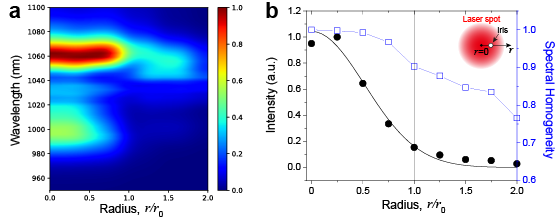


**Figure S16.** (a) The radial spectral distribution of the SCG generated by 1 mJ pulses under the resonant conditions (b) The intensity profile and the spectral homogeneity over the beam profile. Inset: The illustration of the spatial filtering experiment.

**Reference**

[S1] L. Bergé, S. Skupin, R. Nuter, J. Kasparian, and J. P. Wolf, Reports Prog. Phys. **70**, 1633 (2007).

[S2] R. Adair, L. L. Chase, and S. A. Payne, Phys. Rev. B **39**, 3337 (1989).

[S3] E. T. J. Nibbering, G. Grillon, M. A. Franco, B. S. Prade, and A. Mysyrowicz, J. Opt. Soc. Am. B **14**, 650 (1997).

[S4] I. H. Malitson and M. J. Dodge, J. Opt. Soc. Am. **62**, 1405 (1972).

[S5] P. E. Ciddor, Appl. Opt. **35**, 1566 (1996).

[S6] A. A. Zozulya, S. A. Diddams, A. G. Van Engen, and T. S. Clement, Phys. Rev. Lett. **82**, 1430 (1999).

[S7] P. Sprangle, J. R. Peñano, and B. Hafizi, Phys. Rev. E **66**, 046418 (2002).

[S8] E. Wigner, Phys. Rev. **40**, 749 (1932).

[S9] J. E. Beetar, S. Gholam-Mirzaei, and M. Chini, Appl. Phys. Lett. **112**, 0 (2018).

[S10] N. Ishii, P. Xia, T. Kanai, and J. Itatani, Opt. Express **27**, 11447 (2019).

[S11] C.-H. Lu, W.-H. Wu, S.-H. Kuo, J.-Y. Guo, M.-C. Chen, S.-D. Yang, and A. H. Kung, Opt. Express **27**, 15638 (2019).

[S12] P. He, Y. Liu, K. Zhao, H. Teng, X. He, P. Huang, H. Huang, S. Zhong, Y. Jiang, S. Fang, X. Hou, and Z. Wei, Opt. Lett. **42**, 474 (2017).

[S13] X. M. Tong and C. D. Lin, J. Phys. B At. Mol. Opt. Phys. **38**, 2593 (2005).

[S14] S. C. Pinault and M. J. Potasek, J. Opt. Soc. Am. B **2**, 1318 (1985).

[S15] J. Weitenberg, A. Vernaleken, J. Schulte, A. Ozawa, T. Sartorius, V. Pervak, H.-D. Hoffmann, T. Udem, P. Russbüldt, and T. W. Hänsch, Opt. Express **25**, 20502 (2017).
